# Sedentary Behaviour and Its Relationship with Early Vascular Ageing in the General Spanish Population: A Cross-Sectional Study

**DOI:** 10.3390/ijerph19095450

**Published:** 2022-04-29

**Authors:** Inés Llamas-Ramos, Rocío Llamas-Ramos, Rosario Alonso-Domínguez, Leticia Gómez-Sánchez, Olaya Tamayo-Morales, Cristina Lugones-Sánchez, Emiliano Rodríguez-Sánchez, Luis García-Ortiz, Manuel A. Gómez-Marcos

**Affiliations:** 1 Institute of Biomedical Research of Salamanca (IBSAL), Primary Care Research Unit of Salamanca (APISAL), 37005 Salamanca, Spain; rociollamas@usal.es (R.L.-R.); rosa90alonso@hotmail.com (R.A.-D.); leticiagmzsnchz@gmail.com (L.G.-S.); olayatm@usal.es (O.T.-M.); crislugsa@gmail.com (C.L.-S.); emiliano@usal.es (E.R.-S.); lgarciao@usal.es (L.G.-O.); magomez@usal.es (M.A.G.-M.); 2 Department of Nursing and Physiotherapy, Universidad de Salamanca, 37007 Salamanca, Spain; 3 Health Service of Castilla and Leon (SACyL), 37005 Salamanca, Spain; 4 University Hospital of Salamanca, 37007 Salamanca, Spain; 5 Department of Medicine, Universidad de Salamanca, 37007 Salamanca, Spain; 6 Department of Biomedical and Diagnostic Sciences, Universidad de Salamanca, 37007 Salamanca, Spain

**Keywords:** sedentary behaviour, early vascular ageing, normal vascular ageing, healthy vascular ageing, general population

## Abstract

Sedentary behaviour is associated with a greater predisposition to developing cardiovascular diseases. The aim of the study was to analyse the relationship between sedentary time and early vascular ageing. A total of 501 participants (49.70% men) were recruited through random sampling stratified by age group and sex. Vascular ageing was evaluated considering three criteria: (1) the vascular ageing index (VAI); (2) the carotid–femoral pulse wave velocity (cfPWV) 10th and 90th percentiles of the reference values in the European population by age; and (3) the Framingham’s heart age. The carotid intima–media thickness was measured using a Sonosite Micromaxx ultrasound, the presence of peripheral artery disease was assessed by calculating the ankle–brachial index using a VaSera VS-1500, and the cfPWV was measured with a SphygmoCor^®^ device. Weekly sedentary hours were evaluated through a sitting time questionnaire. The average age of the population was 55.90 ± 14.24 years. The men spent more hours sitting per week (47.6 ± 16.6 vs. 36.8 ± 17.3 h/W), at work (16.7 ± 16.2 vs. 9.73 ± 14.9 h/W), and watching TV (21.6 ± 12.5 vs. 18.7 ± 11.9 h/W). In the logistic regression analysis, the individuals with early vascular aging (EVA), with respect to those with healthy vascular aging (HVA), spent more hours sitting per week (OR = 1.03 vs. OR = 1.02; *p* < 0.05) and watching TV (OR = 1.03 vs. OR = 1.03; *p* < 0.05), using the criteria of the European guideline and VAI, and more hours sitting when commuting (OR = 1.04; *p* < 0.05), using Framingham’s heart age to define EVA. The results of this study indicate that sedentary time is associated with early vascular ageing. Therefore, reducing sedentary time would improve vascular health.

## 1. Introduction

Sedentary behaviour and physical inactivity have become the “disease” of the 21st century, causing a large number of chronic conditions [1,2]. The relationship between sedentary lifestyles and the prevalence of cardiometabolic disorders has been demonstrated [3,4,5]. Sedentary behaviour is associated with greater predisposition to develop cardiovascular diseases, and it is one of the main risk factors of coronary disease [6,7,8,9,10,11]. It is currently estimated that 55–70% of daily activities, without considering the time spent sleeping, are considered sedentary [3,12,13,14], and sedentary time increases with age [13,15,16].

Vascular ageing is influenced by the parameters of vascular structure and function, reflecting the dissociation between the chronological age and biological age of the main arteries, whose alteration leads to the appearance of cardiovascular events [17,18,19,20,21].

In recent years, several epidemiological studies have been conducted to unravel the determining factors of vascular ageing, and the results are very relevant, as they show a stronger correlation with morbimortality by cardiovascular diseases than biological ageing [17,18,22]. Among these determining factors of ageing [23,24,25,26], numerous studies have established that physical activity is a great ally against ageing. In this sense, Niiranen et al., based on the studies of Framingham [27] and Gómez Sánchez et al. [28], concluded that individuals who perform physical activity present lower vascular ageing. In addition, there are articles that argue that moderate-intensity exercise seems to produce higher benefits than low-intensity in the physical wellbeing and quality of life among older people [29], and virtual reality has recently been used to improve the cognitive status and dual task performance of elderly men [30]. However, few studies have analysed the association between vascular ageing and sedentary time, both global time and that dedicated to different activities of daily living. Nevertheless, some studies report that, in sedentary adults, ageing is associated with greater stiffness of the main elastic arteries, with a more deficient vascular endothelial function and greater mortality [23,31]. In line with these data, the EVIDENT study found a relationship between sedentary time (specifically the hours spent watching TV) and greater arterial stiffness [32].

Finally, we cannot forget the importance of vascular ageing in brain health. Cerebrovascular events increase the risk of vascular dementia between 3.5 and 4.7 times, the second cause of dementia after Alzheimer’s disease, responsible for 15–30% of dementia cases [33]. In addition, vascular dementia can coexist with other diseases such as Alzheimer’s disease [34]. Vascular dementia is caused by diseases of the small blood vessels that play an important role in vascular ageing [35].

The main hypothesis of this study is that total sedentary time is related to early vascular ageing (EVA) and may differ depending on the type of sedentary activity.

Therefore, the aim of this study was to analyse the association between sedentary time and EVA analysed with different criteria in a Spanish sample with no history of diagnosed cardiovascular disease.

## 2. Materials and Methods

### 2.1. Study Design

This is a cross-sectional, descriptive, observational study with a sample of individuals recruited in the study entitled *Association between different risk factors and early vascular ageing (EVA study)*. The study was registered at ClinicalTrials.gov with the identifying code NCT02623894 [36].

### 2.2. Participants

The study was conducted at the Salamanca Primary Care Research Unit. The reference population was 43,946 people between the ages of 35 and 75 residing in the capital of Salamanca and treated in the public health system. From the database of people treated in five health centres, we carried out a random sampling with replacement stratified by age group (35, 45, 55, 65, and 75 years) and sex, selecting 501 individuals, with approximately 100 in each group of the 5 age groups and half of each sex. The recruitment was carried out between June 2016 and November 2017. Figure S1 of the Supplementary Material shows the flow chart of the individuals included in the study. A detailed description of the procedures of the study has been previously published, as well as the inclusion and exclusion criteria and the response rate [36,37].

The inclusion criteria were age between 35 and 75 years and having signed the informed consent. The exclusion criteria were terminal condition, unable to travel to the health centres, history of cardiovascular disease, glomerular filtration rate below 30%, chronic inflammatory disease or acute inflammatory process in the past three months, and patients treated with oestrogens, testosterone, or growth hormones.

To estimate the power of the sample, we considered the difference in the main variable, total weekly sedentary time, between the EVA group (89 subjects) and the rest (412 subjects), with the European population cut points. The mean sedentary time of the first group was 46.62 h/week and of the second 41.13 h/week, with a common SD of 16.93 h. Therefore, for a bilateral contrast and assuming an alpha risk of 0.05, the estimated power was 80%.

### 2.3. Ethical Considerations

The study was approved by the Clinical Research Ethics Committee of the Health Area of Salamanca on 4 May 2015, with registration number PI15/01039. Before initiating the study, all participants were informed about the objectives of the study and signed an informed consent form. The entire study complied with the principles of the Declaration of Helsinki [38] and the standards of the WHO for observational studies.

### 2.4. Variables and Measurement Instruments

Two healthcare professionals, who were trained before the beginning of the study, collected the data, following a standardised protocol.

#### 2.4.1. Measurement of Sedentary Time

A sitting time questionnaire was used to evaluate the sedentary weekly hours by asking the participants about the hours they spent sitting per day, differentiating between the hours spent sitting when commuting, at work and watching TV, also considering working and non-working days [39,40].

#### 2.4.2. Criteria to Define Vascular Ageing

Firstly, using the criteria established in the 2018 clinical practice guideline of European Societies of Hypertension and Cardiology for the treatment of arterial hypertension [20], the 59 individuals who presented vascular injury in the carotid arteries or peripheral artery disease were classified in the early vascular ageing (EVA) group. 

To classify the remaining 442 individuals, the following three criteria were used:

1. The vascular ageing index (VAI) [19], calculated from the carotid–femoral pulse wave velocity (cfPWV) and the carotid intima–media thickness (cIMT), with the following formula: VAI = (log (1.09) × 10 cIMT + log (1.14) cfPWV) × 39.1 + 4.76. The participants were ordered in percentiles of VAI, by age and sex, classifying them as: EVA, if VAI ≥ 90th percentile; normal vascular ageing (NVA), if 10th percentile < VAI < 90th percentile; and healthy vascular ageing (HVA), if VAI ≤ 10th percentile.
2. The 10th and 90th percentiles of cfPWV, by age, of the reference values published by Boutouyrie P in the European population [41], were used to classify the participants as EVA if cfPWV ≥ 90th percentile; NVA, if 10th percentile < cfPWV < 90th percentile; and HVA, if cfPWV ≤ 10th percentile.
3. Coronary age was estimated with the D’Agostino version of the scale of cardiovascular risk (CVR) based on the study of Framingham [42]. Distributing the individuals following the criterion established by Appiah et al. [43], we classified them as EVA if the difference between chronological age and heart age was greater than 5 years, NVA if the difference was 5 years, and HVA if the difference was lower than 5 years.

#### 2.4.3. Measurement of Vascular Injury

The common carotid artery was measured after examining a 10 mm longitudinal section at a distance of 1 cm from the fork, and measurements were taken on the proximal and distal walls [44]. cIMT was measured using a Sonosite Micromaxx ultrasound device (Sonosite Inc., Bothell, WA, USA), with Sonocal software. The presence of peripheral artery disease was assessed by calculating the ankle–brachial index using VaSera VS-1500 (Fukuda Denshi, Denshi Co., Ltd., Tokyo, Japan), following the criteria of the 2018 ESC/ESH Guidelines for diagnosing and treating hypertension to establish the presence of vascular injury [20].

#### 2.4.4. Measurement of Arterial Stiffness

The carotid and femoral pulse waves were recorded, estimating the delay time compared to the electrocardiogram ECG wave to calculate the pulse wave velocity. The distances were recorded using a measuring tape, from the sternal notch to the point where the sensor was placed on the carotid and femoral arteries [45]. The pulse wave was recorded by flattening tonometry, using a sensor on the radial artery; with a mathematical transformation, the device estimates the aortic pulse wave. cfPWV was measured with the participant in the supine position, using a SphygmoCor^®^ device (AtCor Medical Pty Ltd., Head Office, West Ryde, Australia) [45].

The analysis of the rest of the variables used in this study was conducted following the previously published protocols [36].

### 2.5. Statistical Analysis

Continuous variables such as age, hours of sedentary lifestyle per week, and criteria to define ageing are shown as mean ± standard deviation and to calculate the difference between men and women, we used Student’s *t*-test. The analysis of the differences in sedentary time between subjects classified as HVA, NVA, and EVA was performed via analysis of variance (ANOVA). A post hoc analysis to analyse the differences between more than two groups was performed with the least significant difference test. The categorical variables represented, such as the presence of the different cardiovascular risk factors, are shown as a number and percentage, and to estimate the differences between the sexes the χ^2^ test was used. To analyse the association of the indices of sedentary time with vascular ageing, four logistic regression models were performed, one for each of the sedentary times studied. HVA and EVA were used as dependent variables, coding them as HVA = 0 and EVA = 1. The four measurements of sedentary behaviour analysed in this study were used as independent variables. The model was repeated with the three classifications of vascular ageing using VAI, the cfPWV of the European guideline, and the difference with Framingham’s heart age. In all the logistic regression models, we used age and sex (0 = woman, 1 = man) as adjustment variables. In the hypothesis test, an α risk of 0.05 was established as the limit of statistical significance. All analyses were performed using the statistical software SPSS for Windows, v.25.0 (IBM Corp, Armonk, NY, USA).

## 3. Results

### 3.1. Baseline Characteristics

The characteristics of the individuals included in the study regarding the cardiovascular risk factors, consumption of pharmaceuticals, sedentary times, and the measurements of vascular structure and function globally and by sex are described in Table 1. The average age was 55.90 ± 14.24 years. The estimated heart age was −2.98 ± 10.13 lower than the chronological age (men: −0.92 ± 10.21; women: −5.01 ± 9.65). The percentage of individuals diagnosed with arterial hypertension (32.9% vs. 25.8%) and type 2 diabetes mellitus (10.5% vs. 4.8%) was greater in men than in women. The men spent more hours sitting per week than the women globally (47.6 vs. 36.8 h/W), at work (16.7 vs. 9.7 h/W) and watching TV (21.6 vs. 18.7 h/W). The parameters of vascular structure and function were greater in men than in women. 

**Figure 1:**
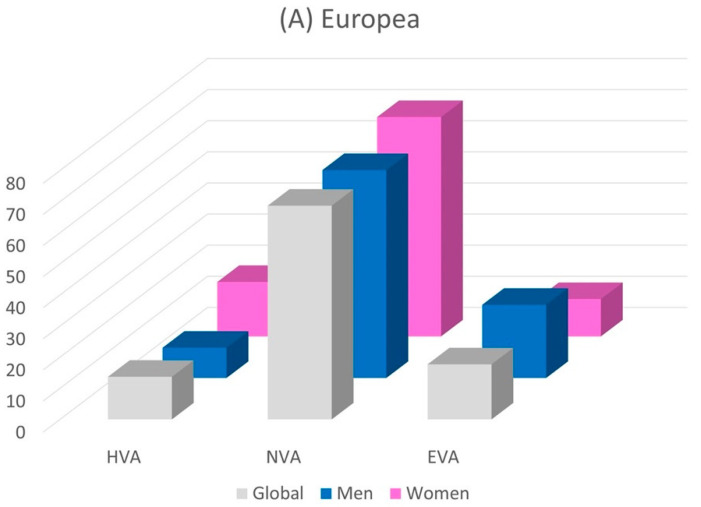

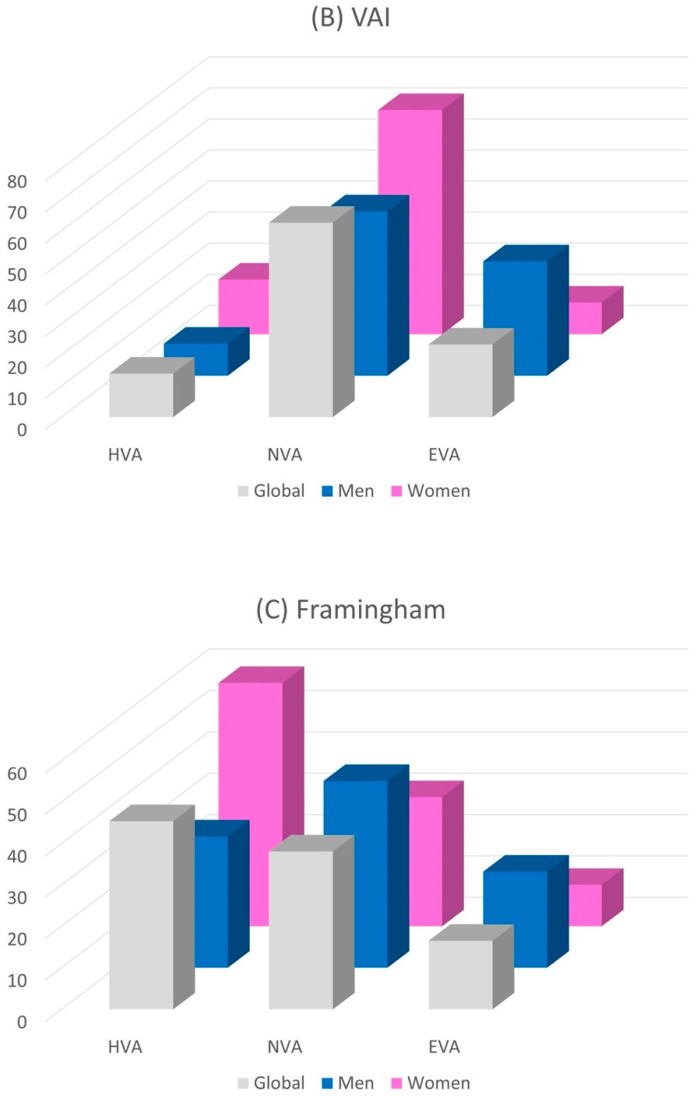
Prevalence in percentage of subjects with HVA, NVA and EVA by sex using the three criteria: cfPWV (**A**), VAI (**B**), and Framingham heart age (**C**). EVA, early vascular aging; HVA, healthy vascular aging; NVA, normal vascular aging; cfPWV, carotid to femoral pulse wave velocity; VAI, vascular aging index.

Figure 1 shows, globally and by sex, the percentage of individuals distributed according to the degree of ageing obtained using the three criteria.

### 3.2. Relationship between Sedentary Time and Vascular Ageing

Table 2 shows the mean values of the sedentary hours per week in the different activities in individuals classified as HVA, NVA and EVA, according to the three criteria used to classify the participants based on the degree of vascular ageing. The increase in the total numbers of sedentary hours and hours watching TV are related to the deterioration of vascular ageing. 

Table 3 shows the differences in the sedentary hours between the individuals classified as HVA and EVA. The individuals classified as HVA spent fewer hours per week sitting and watching TV than those classified in the EVA group.

### 3.3. Association of Sedentary Time with Vascular Ageing 

**Figure 2:**
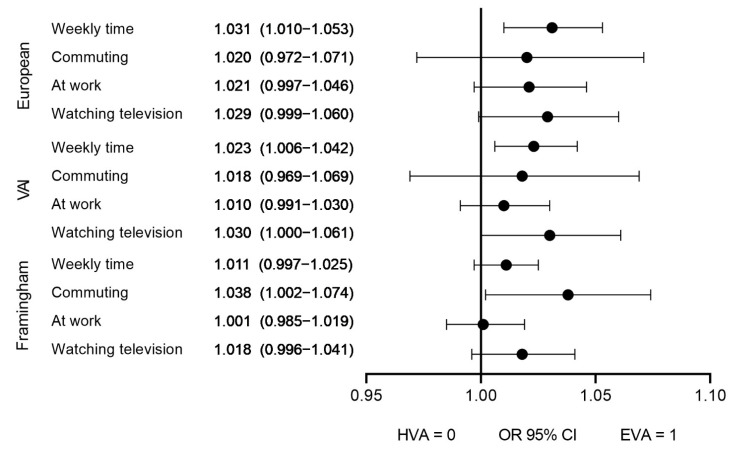
Logistic regression analysis. Association between sedentary time with vascular aging using the three criteria: cfPWV, VAI and Framingham heart age. Independent variables: weekly time, commuting time, at work time, and watching television time. Dependent variables: HVA and EVA (HVA = 0 y EVA = 1). HVA, healthy vascular aging; EVA, early vascular aging; cfPWV, carotid to femoral pulse wave velocity; VAI, vascular aging index. IC 95%: confidence interval of 95%.

In the logistic regression analysis controlled for age and sex, the EVA individuals spent more hours per week sitting compared to the HVA participants, with odds ratio (OR) = 1.031 (CI 95% 1.010 to 1.053) following the criteria of the European guideline and OR = 1.023 (CI 95% 1.006 to 1.042) following the VAI criteria. Regarding the hours spent sitting watching TV, the EVA individuals, compared to the HVA participants, obtained OR = 1.029 (CI 95% 0.999 to 1.060) according to the European guideline and OR = 1.030 (CI 95% 1.000 to 1.061) according to VAI. The heart age obtained a stronger correlation with sedentary time when commuting, with OR = 1.038 (CI 95% 1.002 to 1.074) (Figure 2).

## 4. Discussion

This study analysed the association of sedentary time with vascular ageing globally and in different activities of daily living. It was observed that the increase in sedentary hours per week, globally and when watching TV, was associated with vascular ageing estimated through the European guideline and VAI, whereas the number of hours spent sitting when commuting was associated with vascular ageing estimated through coronary age (Framingham) in the general Spanish population aged 35–75 years and with no history of cardiovascular disease.

Moreover, the results showed that the men spent more hours sitting per week, globally, at work and when watching TV, with respect to the women. These results are in line with those published in previous studies. With respect to the women, the men spent more hours sitting and watching TV, using computers or playing videogames [46]. A study conducted in British men [47] showed that over 72% of the time of the participants was sedentary. Furthermore, studies carried out in Europe, the USA, and Australia showed that adults spent half of their working hours sitting (4.2 h/day) and around 2.9 h/day sitting during their leisure time [48]. Moreover, prolonged sedentary time has been associated with other CVR [49,50] regardless of physical activity. Thus, a study conducted in an Australian population associated every additional hour of sedentary time with a 4% and 5% higher probability of developing CVR factors in women and men, respectively [49].

The relationship between the means of vascular function and sedentary time has been described by other authors, who evaluated the cardiovascular function in sedentary adults when they carried out different types of physical activity [51,52]. Thus, the EVIDENT study showed a correlation between the sedentary time and some parameters of arterial ageing, such as ambulatory arterial stiffness index (AASI) [53]; although, not all authors have found an association between physical activity and vascular function [54].

To the best of our knowledge, there have been no studies that have analysed the association between vascular ageing and sedentary time (neither total sedentary time nor that spent in different activities of daily living). However, extensive evidence shows a relationship between sedentary behaviour and worse cardiovascular health [49,50,55]. Nevertheless, the studies that have analysed the correlation between physical activity and arterial stiffness show contradicting results. Thus, while Slivovskaja et al. [56] did not find a relationship between arterial stiffness and walking, doing house chores, gardening, or DIY activities, Ahmadi et al. [57] reported that limiting the sedentary time was associated with a slower age-related progression of aortic stiffness. This finding would support the results obtained in the present study, which suggest that individuals who spend more hours sitting have a higher probability of being classified as EVA.

In this sense, several studies have shown that the time spent watching TV is related to worse cardiovascular health [58,59], and Wennman et al. [55] found that the risk of developing a cardiovascular disease estimated with Farmingham’s equation was greater for the men who spent over four hours watching TV and for the women who spent two to three hours watching TV. Furthermore, another study that analysed the hours spent in front of the computer only found a correlation in men [55]. These results support those reported in the present study and suggest that the distribution of the sedentary hours in the activities of daily living can be different depending on sex.

### Limitations

The main limitation of this study is the cross-sectional analysis of the data, which does not allow for inferring causality. Secondly, the sample consisted of an urban population of a Spanish city aged 35–75 years; thus, no individuals outside of this age range were included. Lastly, the sedentary time was evaluated through a self-reported questionnaire, which is a subjective measure.

## 5. Conclusions

The sedentary time is associated with early vascular ageing. Therefore, reducing sedentary time would improve vascular health.

## Figures and Tables

**Table 1:** General characteristics of the subjects included globally and by sex.

	Global (501)	Men (249)	Women (252)	*p* Value
**Conventional Risk Factors**				
Age, years	55.90 ± 14.24	55.95 ± 14.30	55.85 ± 14.19	0.935
Framingham’s heart age, years	52.98 ± 17.31	55.09 ±16.53	50.92 ± 17.85	0.007
Heart age–chronological age, years	−2.98 ± 10.13	−0.92 ± 10.21	−5.01 ± 9.65	<0.001
Smoker, n (%)	90 (18.00)	49 (19.70)	41.00 (16.30)	0.190
Hypertensive, n (%)	147 (29.30)	82 (32.90)	65 (25.80)	<0.001
Antihypertensive drugs, n (%)	96 (19.20)	50 (20.10)	46 (18.30)	0.650
Dyslipidemia, n (%)	191 (38.10)	95 (38.10)	96 (38.20)	0.989
Lipid–lowering drugs, n (%)	102 (20.40)	49 (19.70)	53 (21.00)	0.396
Diabetes mellitus, n (%)	38 (7.60)	26 (10.50)	12 (4.80)	0.012
Hypoglycemic drugs, n (%)	35 (7.00)	23 (9.20)	12 (4.80)	0.055
Obesity, n (%)	94 (18.80)	42 (16.90)	52 (20.60)	0.304
**Sedentary Time**				
Weekly time, h/W	42.18 ± 16.93	47.60 ±16,58	36.76 ±17.29	<0.001
Commuting, h/W	3.31 ± 7.48	3.64 ± 8.35	2.99 ± 6.62	0.335
At work, h/W	13.19 ± 15.52	16.66 ± 16.16	9.73 ± 14.89	<0.001
Watching television, h/W	20.13 ± 12.18	21.55 ± 12.45	18.72 ± 11.91	0.010
**Vascular Assessment**				
c-IMT (mm)	0.682 ± 0.109	0.699 ± 0.115	0.665 ± 0.100	0.001
cfPWV (m/s)	8.17 ± 2.49	8.58 ± 2.74	7.77 ± 2.24	<0.001
VAI	61.25 ± 12.64	63.47 ± 13.75	59.04 ± 11.54	<0.001

Values are means ± standard deviations for continuous data and number and proportions for categorical data. c-IMT, carotid intima–media thickness; cfPWV, carotid to femoral aortic pulse wave velocity; VAI, vascular aging index; h/W, hours/week. *p* value: differences between men and women.

**Table 2:** Characteristics of participants with healthy vascular ageing, normal and early vascular ageing according to the three criteria.

	HVA	NVA	EVA	*p* Value
**Using 10th Percentile and 90th Percentile of European Population Cut Points**				
Weekly time, h/W ^¥,#^	38.78 ± 18.53	41.60 ± 17.91	46.62 ± 15.95	0.015
Commuting, h/W	2.78 ± 6.07	3.43 ± 7.54	3.37 ± 8.60	0.808
At work, h/W	12.41 ± 14.39	13.09 ± 16.32	14.17 ± 15.48	0.776
Watching television, h/W ^¥,#^	18.29 ± 11.66	19.56 ± 12.08	23.46 ± 12.47	0.012
**Using 10th Percentile and 90th Percentile of VAI**				
Weekly time, h/W ^¥,#^	39.69 ± 19.94	40.82 ± 17.42	47.05 ± 16.43	0.003
Commuting, h/W	2.62 ± 6.52	3.60 ± 7.76	3.07 ± 7.69	0.574
At work, h/W	13.95 ± 18.05	12.51 ± 15.45	15.23 ± 16.01	0.282
Watching television, h/W ^¥,#^	18.08 ± 9.77	19.49 ± 12.23	22.76 ± 12.90	0.017
**Using Framingham’s Heart Age**				
Weekly time, h/W *	39.27 ± 17.17	44.88 ± 16.95	42.74 ± 20.09	0.005
Commuting, h/W	2.82 ± 5.34	3.29 ± 8.12	4.66 ± 10.70	0.168
At work, h/W	12.34 ± 15.25	14.40 ± 16.19	11.88 ± 16.62	0.325
Watching television, h/W *^,#^	18.34 ± 11.22	21.74 ± 12.52	21.60 ± 13.94	0.010

Values are means ± standard deviations for continuous data. Differences among groups: continuous variables analysis of variance and post hoc using the least significant test (LSD). HVA, healthy vascular aging; NVA, normal vascular aging; EVA, early vascular aging; h/W, hours/week. * *p* value < 0.05 between HVA and NVA. ^¥^ *p* value < 0.05 between NVA and EVA. ^#^ *p* value < 0.05 between HVA and EVA.

**Table 3:** Differences between subjects with HVA and subjects with EVA.

	Difference	95% CI
**Using 10th Percentile and 90th Percentile of European Population Cut Points**		
Weekly time, h/W	−7.840	(−13.413–−2.267)
Commuting, h/W	−0.589	(−2.912–1.733)
At work, h/W	−1.755	(−6.500–2.990)
Watching television, h/W	−5.173	(−9.009–−1.338)
**Using 10th Percentile and 90th Percentile of VAI**		
Weekly time, h/W	−7.365	(−13.041–−1.689)
Commuting, h/W	−0.451	(−2.564–1.661)
At work, h/W	−1.287	(−6.537–3.964)
Watching television, h/W	−4.680	(−8.020–−1.339)
**Using Framingham’s Heart Age**		
Weekly time, h/W	−3.467	(−8.409–−1.474)
Commuting, h/W	−1.840	(−4.289–0.608)
At work, h/W	0.457	(−3.694–4.608)
Watching television, h/W	−3.263	(−6.651–−0.126)

HVA, healthy vascular aging; EVA, early vascular aging; VAI, vascular aging index; h/W, hours/week; CI, confidence interval.

## Data Availability

The data underlying this article will be shared on reasonable request to the corresponding author.

## Supplementary Materials

The following supporting information can be downloaded at: https://www.mdpi.com/article/10.3390/ijerph19095450/s1, Figure S1: Study design. Patient selection and inclusion flowchart.
