# Supplementary figures and images for: Sedentary Behaviour and Its Relationship with Early Vascular Ageing in the General Spanish Population: A Cross-Sectional Study
Source: Int J Environ Res Public Health. 2022 Apr 29;19(9):5450. doi: 10.3390/ijerph19095450 (PMC9101612; doi:10.3390/ijerph19095450)

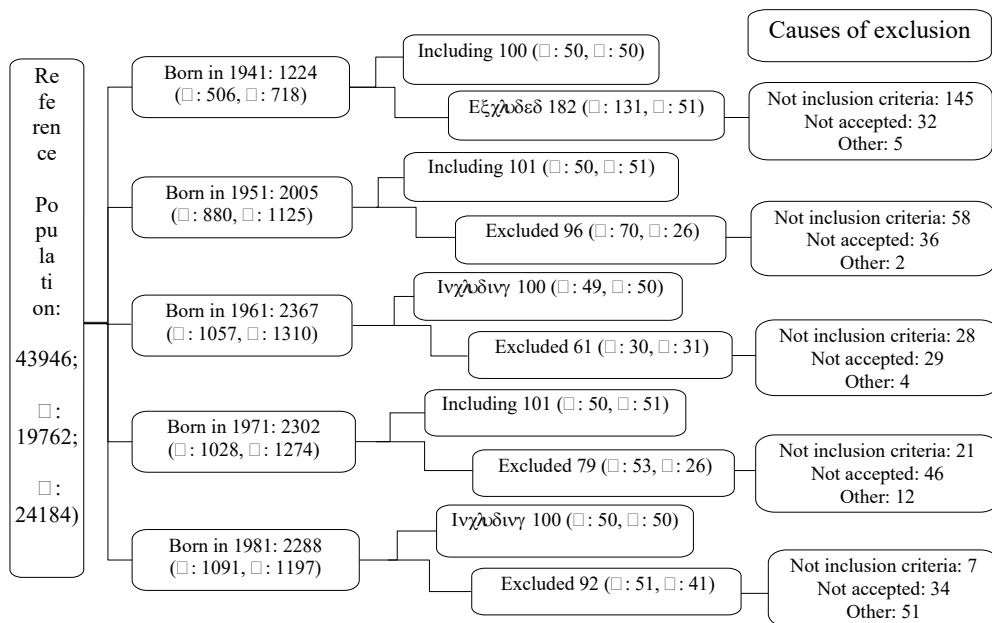

**Figure S1.** Study design. Patient selection and inclusion flowchart.

Supplement: Supplementary file 1 [file ijerph-19-05450-s001.zip › ijerph-1659136-supplementary.pdf]
